# Significant Tumor Regression after Neoadjuvant Chemotherapy in Gastric Cancer, but Poor Survival of the Patient? Role of MHC Class I Alterations

**DOI:** 10.3390/cancers15030771

**Published:** 2023-01-26

**Authors:** Theresa Hiltner, Noémi Szörenyi, Meike Kohlruss, Alexander Hapfelmeier, Anna-Lina Herz, Julia Slotta-Huspenina, Moritz Jesinghaus, Alexander Novotny, Sebastian Lange, Katja Ott, Wilko Weichert, Gisela Keller

**Affiliations:** 1 Institute of Pathology, TUM School of Medicine, Technical University of Munich, 81675 Munich, Germany; 2 Institute of Medical Informatics, Statistics and Epidemiology, Technical University of Munich, 81675 Munich, Germany; 3 Institute of General Practice and Health Services Research, School of Medicine, Technical University of Munich, 81675 Munich, Germany; 4 Institute of Pathology, University Hospital Marburg, 35043 Marburg, Germany; 5 Department of Surgery, TUM School of Medicine, Technical University of Munich, 81675 Munich, Germany; 6 II. Medizinische Klinik, Klinikum rechts der Isar, Technical University of Munich, 81675 Munich, Germany; 7 Department of Surgery, Klinikum Rosenheim, 83022 Rosenheim, Germany; 8 German Cancer Consortium (DKTK), Partner Site Munich, Institute of Pathology, 81675 Munich, Germany

**Keywords:** adenocarcinoma, gastric, gastroesophageal junction, prognosis, tumor regression, neoadjuvant chemotherapy, HLA, B2M, allelic imbalance, loss of heterozygosity

## Abstract

**Simple Summary:**

The major histocompatibility complex (MHC) class I genes, encompassing the human leukocyte antigen (HLA) class I and the beta-2 microglobulin (B2M) genes, play a key role in neoantigens presentation to the immune system. We analyzed allelic imbalance (AI) of the respective chromosomal regions by multiplex PCRs using microsatellite markers in biopsies of 158 patients with gastric/gastroesophageal adenocarcinoma before neoadjuvant platinum/fluoropyrimidine chemotherapy (CTx) for an association with clinical outcome of the patients. AI with no marker was significantly associated with response or survival. However, subgroup analysis revealed interesting differences. Of note, AI at markers of the HLA region was associated with a decreased survival only in responding but not in non-responding patients. No associations were observed for B2M markers. Our results underline the importance of intact neoantigen presentation specifically for responding patients and may help explain an unexpectedly poor survival of a patient despite significant tumor regression after neoadjuvant CTx.

**Abstract:**

We aimed to determine the clinical and prognostic relevance of allelic imbalance (AI) of the major histocompatibility complex (MHC) class I genes, encompassing the human leukocyte antigen (HLA) class I and beta-2 microglobulin (B2M) genes, in the context of neoadjuvant platinum/fluoropyrimidine chemotherapy (CTx). Biopsies before CTx were studied in 158 patients with adenocarcinoma of the stomach or gastroesophageal junction. The response was histopathologically evaluated. AI was detected by multiplex PCRs analysis of four or five microsatellite markers in HLA and B2M regions, respectively. AI with no marker was significantly associated with response or survival. However, subgroup analysis revealed differences. AI at marker D6S265, close to the HLA-A gene, was associated with an obvious increased risk in responding (HR, 3.62; 95% CI, 0.96–13.68, *p* = 0.058) but not in non-responding patients (HR, 0.92; 95% CI, 0.51–1.65, *p* = 0.773). Markers D6S273 and D6S2872 showed similar results. The interaction between AI at D6S265 and response to CTx was significant in a multivariable analysis (*p* = 0.010). No associations were observed for B2M markers. Our results underline the importance of intact neoantigen presentation specifically for responding patients and may help explain an unexpectedly poor survival of a patient despite significant tumor regression after neoadjuvant platinum/fluoropyrimidine CTx.

## 1. Introduction

Despite the declining incidence of gastric cancer, the disease is still the third leading cause of cancer death worldwide [1]. Platinum/5-fluorouracil-based pre-/perioperative chemotherapy was shown to increase survival of advanced gastric- or gastroesophageal cancer patients, and the inclusion of docetaxel into the therapeutic regimens then further improved the prognosis of the patients [2,3].

Response to cytotoxic chemotherapy is evaluated based on the histopathological determination of the tumor’s regression using various scoring systems [4,5]. According to Becker’s classification, the responses are classified into three grades. Tumor regression grade 1 (TRG1) corresponds to no or less than 10%, TRG2 to 10–50%, and TRG3 to >50% of residual tumor cells in the resected specimen [5]. This grading system is associated with overall survival and is considered a valuable prognostic factor for predicting treatment outcomes [6,7,8,9].

In a recent study, we demonstrated an association of a high degree of chromosomal instability (CIN) in tumor biopsies before neoadjuvant chemotherapy with good histopathological tumor regression, which, however, did not translate into a significant survival benefit in this specific subgroup of patients [10]. CIN is one of the major hallmarks of many solid tumors, including gastric carcinomas [10,11]. A considerable interplay between the effects of chemotherapeutic agents and the tumor microenvironment has been described, and beyond that, CIN has been associated with immune evasion and immunological “cold” tumors in various tumor entities [12,13,14]. Thus, we asked whether the loss of genes or chromosomal regions, which are critical for antigen presentation, is of particular importance in this context. 

The major histocompatibility complex (MHC) class I presents specific tumor neoantigens to cytotoxic T-lymphocytes and is essential for provoking an antitumor immune response, and loss of MHC class I is associated with worse clinical outcomes in various cancer types [15,16,17,18]. MHC class I consists of a heavy alpha chain encoded by three human leukocyte antigen (HLA) genes (HLA-A, HLA-B, and HLA-C) located on chromosome 6p21 and a light chain encoded by the beta-2 microglobulin (B2M) gene located on chromosome 15q21 [15,16,17].

The aim of our study was to clarify whether allelic imbalance (AI) suggestive of a loss of heterozygosity (LOH) at these specific chromosomal regions in tumor biopsies before neoadjuvant treatment may have an impact on patient survival despite significant histopathological tumor regression. 

## 2. Material and Methods

### 2.1. Patients and Chemotherapy

Biopsies of gastric adenocarcinomas, including tumors of the gastroesophageal junction (AEG II and AEG III, according to Siewert and Stein [19]) before neoadjuvant CTx from 158 patients, were analyzed. Patients were treated at the Department of Surgery of the Technical University of Munich between 1995 and 2017. Inclusion criteria for the present study were treatment with fluoropyrimidine/cis- or oxaliplatin-based neo-adjuvant chemotherapy (Supplementary Table S1) and the availability of DNA or paraffin blocks from tumor and non-tumorous tissues. Tumors with high microsatellite instability (MSI-H) were excluded as described [10] (Figure S1). The patient characteristics are summarized in Table 1.

### 2.2. Response Evaluation

The response was classified according to Becker into three tumor regression grades (TRG): TRG1, TRG2, and TRG3, which corresponded to <10%, 10–50%, and >50% residual tumor cells present in the resected specimen, respectively [6]. Patients with TRG1 were classified as responders, whereas those with TRG2 and TRG3 were classified as non-responders.

### 2.3. Follow-Up and Overall Survival

Follow-up was performed as described, and the overall survival (OS) of the patients was defined as the time between the date of surgery and death by any cause [20].

### 2.4. Analysis of Allelic Imbalance

**Figure 1:**
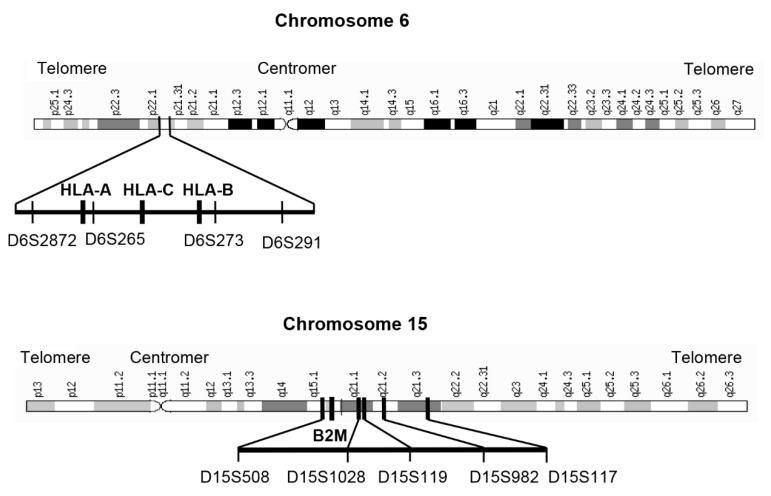
Chromosomal localization of the microsatellite markers. Localization of the microsatellite markers at chromosome 6p21 relative to the HLA-A, HLA-B, and HLA-C genes and of the microsatellite markers on chromosome 15q21 relative to the B2M gene region is shown.

DNA from paired tumor and non-tumor formalin-fixed paraffin-embedded (FFPE) tissues were isolated as described [21,22]. Allelic imbalance (AI) was considered as a surrogate for loss of heterozygosity (LOH) and was detected using two microsatellite-based multiplex PCR assays encompassing four or five microsatellite markers spanning the chromosomal regions of the HLA genes on chromosome 6p21 and the B2M gene on chromosome 15q21, respectively. Markers for chromosome 6p21 were D6S291, D6S273, D6S265, and D6S2872, and those for chromosome 15q21 were D15S508, D15S1028, D15S119, D15S982, and D15S117. The relative positions of microsatellite markers on each chromosome are shown in Figure 1. Design and PCR conditions are detailed in the Supplementary Methods [21,23,24]. Primer sequences are summarized in Table S2 [25,26,27,28,29,30]. Individual cut-off values for the definition of AI were determined for each marker as described in the Supplementary Methods [21,24,31]. A representative dot plot related to the cut-off definition is shown in Figure S2. The composition of the primer mixtures and the cut-off values are summarized in Table S3.

### 2.5. Statistical Analyses

The distribution of quantitative and qualitative data is presented by descriptive statistics, such as the median and range or absolute and relative frequencies, respectively. Kaplan–Meier estimates of survival probabilities were compared between patient subgroups defined by clinical characteristics using log-rank tests. Relative risks were estimated using hazard ratios (HRs) from the univariable and multivariable Cox proportional hazard models. Interaction effects were added to these models to assess group differences in relative risks. According to the recommendation of Peduzzi et al. [32], the ratio of the number of variables in the multivariable Cox regression model to the number of events was limited to 1:10. Two-sided Chi-squared tests or Fisher’s exact tests were used to compare relative frequencies. Overall, two-sided exploratory 5% significance levels (two-tailed) were used for hypothesis testing. Respective 95% confidence intervals were calculated for the effects measured. All statistical analyses were performed using SPSS Statistics 27 (IBM Corp., Armonk, NY, USA) and R version 4.0.3 (The R Foundation for Statistical Computing, Vienna, Austria). 

## 3. Results

### 3.1. Frequency of Allelic Imbalance and Association with Clinicopathological Parameters

AI of the four microsatellite markers on chromosome 6p21 was detected in a range from 38.8% to 60.0% and in a range from 56.5% to 67.0% for markers on chromosome 15q21. The results are shown in Table 2. Representative images of AI analysis are shown in Figure S3. 

A statistically significant association was found between the markers D15S1028 and D15S119 and tumor localization. Out of the 72 informative and proximally located tumors, 55 (76.4%) showed an AI compared with 23 of 45 (51%) informative and non-proximally located tumors for marker D15S1028 (*p* = 0.008). The results were similar for marker D15119 (*p* = 0.023). Another significant difference was found between marker D15S508 and resection status. Among the 49 informative and R0-resected patients, 26 (53.1%) showed an AI compared with 11 of 12 (91.7%) R1-resected patients (*p* = 0.019). No statistically significant associations with sex, age, histopathological tumor type, tumor localization, ypT, ypN, metastasis, or resection status were found for any of the other markers (Table S4).

### 3.2. Allelic Imbalances and Response to Neoadjuvant CTx

No significant association was found for AI at any of the microsatellite markers spanning chromosomal region 6p21 or 15q21 with response to neoadjuvant CTx in terms of histopathological tumor regression. A slightly higher frequency of AI was observed for maker D15S117 among the non-responders, as 56 of 91 (61.5%) informative patients showed an AI compared with 18 of 40 (45.0%) informative responding patients (*p* = 0.079). The results are summarized in Table 3.

### 3.3. Allelic Imbalances and Univariable Survival Analysis 

In the study as a whole, no statistically significant association of AI at any of the microsatellite markers with patient survival was observed. A somewhat worse OS was observed in patients with AI at marker D6S2872 (HR, 1.65; 95% CI, 0.93–2.91, *p* = 0.087) and for patients with AI at D15S117 in their tumors (HR, 1.63; 95% CI, 0.97–2.72, *p* = 0.063) (Table S5). 

**Figure 2:**
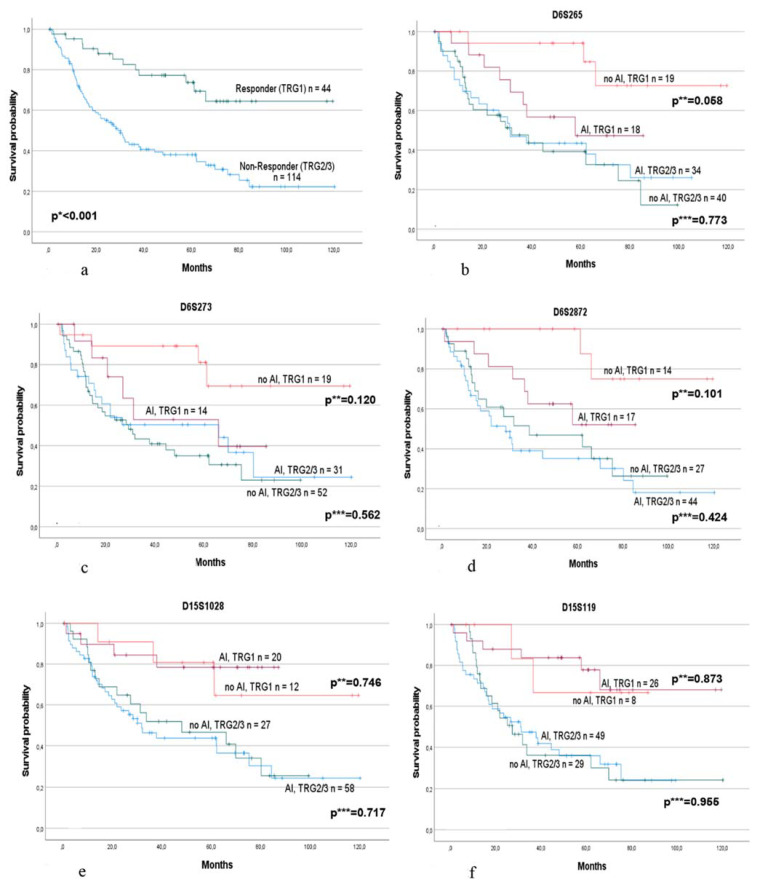
Survival of the responding and non-responding patients alone and in association with AI at specific microsatellite markers of chromosome 6p21 and 15q21. Survival of (**a**) the responding (TRG1) and non-responding (TRG2/3) patients. *p* * value log-rank test. Survival in association with AI (allelic imbalance) or no AI in responding and non-responding patients separately at marker (**b**) D6S265; (**c**) D6S273; (**d**) D6S2872; (**e**) D15S1028; and (**f**) D15S119. *p* ** values: univariable Cox regression for patients with AI and no AI in the responding group. *p* *** values: univariable Cox regression for patients with AI and no AI in the non-responding group.

A separate subgroup analysis of the responding and non-responding patients showed, as expected, a greater difference in survival (*p*_log-rank_ < 0.001) (Figure 2a), and we observed striking differences for specific markers. 

Of note, considering the markers of chromosome 6p21, AI was associated with worse OS in the responding group but not in the non-responding group. In particular, marker D6S265 demonstrated a qualitative difference between the HR of AI in responders (HR, 3.62; 95% CI, 0.96–13.68, *p* = 0.058) compared with non-responders (HR, 0.92; 95% CI, 0.51–1.65, *p* = 0.773), which indicates an almost four-fold increased risk in responders with an AI in their tumors and only a minor decreased risk by AI in non-responders (Figure 2b). Increased risk of AI was also found for markers D6S2872 (HR, 3.73; 95% CI, 9.77–18.03, *p* = 0.101) and D6S273 (HR, 2.73; 95% CI, 0.77–9.71, *p* = 0.120) in the responding group. In contrast, in the non-responding group, only slight differences were observed for D6S2872 (HR, 1.29; 95% CI, 0.69–2.38, *p* = 0.424) and D6S273 (HR, 0.84; 95% CI, 0.47–1.50, *p* = 0.562) (Figure 2c,d). Testing for an interaction effect between AI and response using univariable Cox regression models was noticeable for markers D6S265 (*p* = 0.064) and D6S273 (*p* = 0.098). No obvious differences were found for marker D6S291. 

Regarding the five markers spanning the region of the B2M gene on chromosome 15q21, no obvious differences between patients with or without AI were observed in either the responding or non-responding group. The Kaplan–Meier survival curves are shown for markers D15S1028 and D15S119 (Figure 2e,f). 

All survival data are summarized in Table 4.

Analyses of the clinical variables of our patients for an association with survival revealed UICC stage (HR, 1.86; 95% CI, 1.45–2.39, *p* < 0.001), R-category (HR, 3.40; 95% CI, 2.11–5.50, *p* < 0.001), response to CTx (HR, 0.307; 95% CI, 0.16–0.56, *p* < 0.001), and sex (HR, 0.57; 95% CI, 0.31–1.03, *p* = 0.061) as conspicuous parameters. No statistically significant differences were observed regarding the Laurén classifications, comparing intestinal versus non-intestinal tumor types (HR, 1.34; 95% CI, 0.87–2.07, *p* = 0.180), tumor localization comparing proximal versus non-proximal tumor localizations (HR, 1.27; 95% CI, 0.82–1.98, *p* = 0.180), and comparing age </≥ median (HR, 1.24; 95% CI, 0.81–1.91, *p* = 0.324).

### 3.4. Multivariable Survival Analysis

Multivariable analysis was performed for the marker D6S265, which showed the most conspicuous result in univariable analysis. Analysis including AI, response, the interaction between AI at D6S265 with the response, and clinical factors with prognostic relevance in the univariable analysis as resection status, UICC stage, and sex, revealed the interaction of D6S265 with the response as a statistically significant variable (HR, 0.14; 95% CI, 0.03–0.63, *p* = 0.010) together with UICC stage (HR, 3.0; 95% CI, 1.53–5.88, *p* = 0.014), R-category (HR, 2.16; 95% CI, 1.13–4.15, *p* = 0.020), and sex (HR, 0.38; 95% CI, 0.18–0.83, *p* = 0.015) (Table 5). 

Analysis in the subgroup of the responding and non-responding patients for the marker D6S265 and adjusting for the above-mentioned variables revealed a significantly reduced risk for AI no compared with AI yes in the responding group (HR, 0.23; 95% CI 0.06–0.93, *p* = 0.038), whereas a slightly increased risk was observed in the non-responding group (HR, 1.74; 95% CI, 0.91–3.30, *p* = 0.092). 

## 4. Discussion

In this study, we analyzed the prognostic relevance of AI suggestive of LOH at two chromosomal regions, 6p21 and 15q21, for GC patients treated with neoadjuvant platinum/fluoropyrimidine CTx. The chromosomal regions 6p21 and 15q21 encompass genes coding for proteins of the MHC class I complex, which is a key component of the antigen presentation pathway and is essential for a proper antitumor immune response [15,16,17]. The most interesting finding of our study was the prognostic significance of AI at markers located around the HLA genes on chromosome 6p21, which we found only in the responding, but not in the non-responding group. Although statistical significance was not reached, the strongest evidence was found for marker D6S265, located close to the HLA-A gene, and to a somewhat lesser extent for marker D6S273 near the HLA-B gene and for marker D6S2872 flanking the HLA-A gene towards the telomeric region. Of note, if the tumors specifically demonstrated an AI in these regions, the patients showed a considerably worse prognosis similar to that of the non-responding patients, despite having tumor regression grade 1, and the interaction between AI at D6S265 with response was a statistically significant factor in the multivariable analysis.

Our results are of particular relevance under several aspects.

We found a prognostic difference of AI at the HLA-I genes only for patients who were classified as responding patients based on the measurement of tumor regression but not for non-responding patients. Although statistical significance was not reached and detailed characterization of the tumor microenvironment has not been performed, it is tempting to speculate that our results may indicate that an intact antigen presentation, together with a good response of the tumor cell itself (exerted by appropriate cytotoxic treatment) is required to translate the chemotherapeutic effect into a survival benefit for patients. Complex interactions between chemotherapy-induced cell death and the tumor microenvironment and immune components have been described and are thought to contribute to therapeutic success [12,13]. Thus, our results may underline an intimate interaction between the immune system and tumor cells in this connection. In addition, our findings may suggest the existence of a specific window regarding the mass of the remaining tumor tissue after CTx, which allows for an optimal antitumor immune response. However, if the tumor mass remains or is too high, various mechanisms of immune evasion beyond HLA class I alterations may be relevant [33,34,35,36,37]. Furthermore, an intact antigen presentation machinery and immune response may even be overcharged to evoke a survival benefit for patients with an advanced, chemoresistant tumor.

Another important aspect of our study is that we did not find statistically significant prognostic relevance for markers spanning the region of the B2M gene. The HLA-I gene complex, which encompasses three classical HLA genes in humans, seems to play a major role in this context. Studies analyzing HLA gene expression have shown that a small reduction in the expression level may affect antigen presentation [38]. HLA-I genes are highly polymorphic, which is related to their ability to bind foreign antigens with various affinities, and small differences in the number of specifically available HLA-I alleles were supposed to impair the presentation of neoantigens [36,39]. Thus, this may be related to the prognostic effect we detected for the HLA-I gene region but not for the B2M region.

In our previous study, we found an association between tumors with high CIN and response to neoadjuvant CTx, which, however, did not translate into a survival benefit for the patients [10]. High CIN or aneuploidy has been described as a feature of immune evasion and was suggested to be a marker of resistance to immune-based therapies [14,36]. It has been speculated that a high CIN may interfere with neoantigen loading onto the MHC and thus may exert a direct mechanistic role in resistance to immune-based therapies [14,36]. However, our results indicate that alterations in specific chromosomal regions involved in an adequate antitumor immune response are of major importance in this context and are critical to translating tumor shrinkage into a survival benefit for the patient. 

HLA class I alterations have been suggested to play a crucial role in immunotherapy resistance [16,39]. Our study, which investigated patients treated with platinum/fluoropyrimidine CTx in the neoadjuvant setting, showed that these alterations are also important for therapy regimens based only on cytotoxic chemotherapeutic agents. This may question whether combined platinum/fluoropyrimidine/immunotherapy regimens could lead to substantial benefits for patients with structural HLA class I alterations in their tumors.

We found AI at the markers of the HLA-I gene region on chromosome 6p21 at relatively high frequencies, with up 60% of the tumors demonstrating AI at marker D6S2872 telomeric flanking the HLA-A gene. LOH of the HLA gene region has been described in different tumor types, for example, in 40–58% of non-small cell lung, colorectal, laryngeal, breast, and gastric cancers [25,37,40,41]. We also found a relatively high frequency of AI in the B2M region, with up to 67% of the tumors exhibiting AI for at least one marker. A considerable variation in the prevalence of LOH in various tumor entities has also been reported for this chromosomal region, with 35% to 54% of colon, bladder, and head and neck carcinomas showing loss of this chromosomal region [26,40]. Different methods have been used for the detection of LOH in these studies, and the high frequency we found in our analysis may be related to the use of individual cut-off values for the definition of AI for each marker, which may allow for more sensitive detection of AI. In addition, our method also detects AI due to copy-neutral LOH, which seems to be a common event in various cancers involving chromosome 6p21 [42]. 

Our study has several limitations that must be addressed. One limitation of this study is its retrospective nature. In addition, particularly in the subgroup analysis, a small number of patients were available; therefore, the results must be interpreted with care. Furthermore, we are aware that the patients in our study were not treated with taxane-containing therapy, which is currently used to treat GC patients. Future studies, including patients treated with neoadjuvant chemotherapy with a taxane-containing compound, should be performed to investigate the role of MHC I alterations in the context of specific treatment protocols. However, our results are based on a relatively homogeneously treated patient cohort, which exemplarily may indicate the intimate interaction between the cytotoxic components of this treatment regimen and the immune system and, finally, may contribute to a better understanding of this complex system.

## 5. Conclusions

Our results demonstrate a prognostic effect of AI for marker D6S265 located in the HLA class I gene region, specifically for responders. This may underline the importance of intact neoantigen presentation in this patient group and may help explain a paradoxically unexpected poor survival of a gastric cancer patient despite significant tumor regression after neoadjuvant platinum/fluoropyrimidine CTx.

## Figures and Tables

**Table 1:** Patient characteristics.

Category	Value	*n* (%)
Cases	Total	158 (100)
Age (yr)	Median	62.2
	Range	30.0–81.3
Overall survival (mo) ^a^	Median	48.1
	95% CI	25.7–70.5
Follow-up period (mo)	Median	61.9
	95% CI	53.5–70.3
Sex	Male	123 (77.8)
	Female	35 (22.2)
Tumor localization	Proximal	102 (64.6)
	Non-proximal	56 (35.4)
Laurén classification	Intestinal	77 (48.7)
	Non-intestinal	81 (51.3)
Clinical tumor stage (cT)	T2	9 (5.9)
	T3/4	143 (94.1)
	n/a	6
ypT ^b^	0	8 (5.1)
	1	17 (10.9)
	2	27 (17.3)
	3	82 (52.6)
	4	22 (14.1)
	n/a	2
ypN ^b^	Negative	65 (41.7)
	Positive	91 (58.3)
	n/a	2
Metastasis status	Negative	108 (69.2)
	Positive	48 (30.8)
	n/a	2
yUICC stage ^b^	0	6 (3.9)
	1	26 (16.7)
	2	38 (24.5)
	3	37(23.9)
	4	48 (31.0)
	n/a	3
Resection category	R0	125 (80.1)
	R1	31 (19.9)
	n/a	2
Tumor regression grade (TRG)	1	44 (27.9)
	2	37 (23.4)
	3	77 (48.7)
Responder Non-responder	TRG1	44 (27.8)
	TRG2, TRG3	114 (72.2)

CI, confidence interval; n/a, not available; ^a^ OS was defined as the time between the date of operation and death by any cause; ^b^ TNM and UICC classification according to the 7th Edition of UICC.

**Table 2:** Frequencies of AI.

Marker	AI (*n*)	Informative * Tumors (*n*)	Frequency (%)
**D6S291**	48	104	46.2
**D6S273**	45	116	38.8
**D6S265**	52	111	46.8
**D6S2872**	61	102	60.0
**D15S508**	38	62	61.3
**D15S1028**	78	117	66.7
**D15S119**	75	112	67.0
**D15S982**	69	111	62.6
**D15S117**	74	131	56.5

* Informative refers to patients with a heterozygous genotype in the normal tissue.

**Table 3:** Association of AI with response to neoadjuvant CTx.

	Responder (TRG1)			Non-Responder (TRG2/3)			
Marker	AI (*n*)	Informative * Tumors (*n*)	Frequency (%)	AI (*n*)	Informative * Tumors (*n*)	Frequency (%)	*p*-Value **
**D6S291**	11	30	36.7	37	74	50.0	0.217
**D6S273**	14	33	42.4	31	83	37.3	0.613
**D** **6S265**	18	37	48.6	34	74	50.0	0.788
**D6S2872**	17	31	54.8	44	71	62.0	0.499
**D15S508**	10	19	52.6	28	43	65.1	0.352
**D15S1028**	20	32	62.5	58	85	68.2	0.557
**D15S119**	26	34	76.5	49	78	62.9	0.158
**D15S982**	19	34	55.9	41	77	53.2	0.797
**D15S117**	18	40	45.0	56	91	61.5	0.079

* Informative refers to patients with a heterozygous genotype in the normal tissue. ** Chi-squared test.

**Table 4:** Survival in the subgroups of the responding and non-responding patients and AI.

Marker	Response Status	AI Status	OS Median (Months) (95% CI)	HR (95% CI)	*p*-Value *	*p*-Value Inter-Action **
**D6S291**	Responder	Yes	n.r.	0.57 (0.12–2.84)	0.497	0.715
		No	n.r.	1		
	Non-responder	Yes	31.05 (21.11–40.99)	0.79 (0.44–1.41)	0.424	
		No	28.40 (15.40–41.40)	1		
**D6S273**	Responder	yes	66.00	2.73 (0.77–9.71)	0.120	0.098
		No	n.r.	1		
	Non-responder	Yes	66.10 (10.24–121.96)	0.84 (0.47–1.50)	0.562	
		No	29.31 (15.48–43.15)	1		
**D6S265**	Responder	Yes	57.80	3.62 (0.96–13.68)	0.058	0.064
		No	n.r.	1		
	Non-responder	Yes	31.34 (16.72–45.97)	0.92 (0.51–1.65)	0.773	
		No	31.90 (11.90–51.90)	1		
**D6S2872**	Responder	Yes	n.r.	3.73 (0.77–18.03)	0.101	0.217
		No	n.r.	1		
	Non-responder	Yes	28.40 (17.38–39.42)	1.29 (0.69–2.38)	0.424	
		No	38.70 (0.00–84.79)	1		
**D15** **S508**	Responder	Yes	n.r.	0.71 (0.14–3.53)	0.543	0.698
		No	n.r.	1		
	Non-responder	Yes	25.21 (0.01–50.42)	1.02 (0.43–2.39	0.968	
		No	44.62 (0.00–91.21)	1		
**D15S1028**	Responder	Yes	n.r.	0.78 (0.18–3.49)	0.746	0.663
		No	n.r.			
	Non-responder	Yes	31.90 (18.71–45.09)	1.12 (0.62–2.03)	0.717	
		No	48.10 (2.14–94.06)	1		
**D15S119**	Responder	Yes	n.r.	0.88 (0.18–4.36)	0.873	0.896
		No	n.r.	1		
	Non-responder	Yes	48.10 (2.14–94.06)	0.98 (0.55–1.74)	0.955	
		No	27.40 (15.11–39.69)	1		
**D15S982**	Responder	Yes	n.r.	0.82 (0.25–2.69)	0.744	0.476
		No	66.00	1		
	Non-responder	Yes	30.20 (12.58–47.82)	1.32 (0.74–2.36)	0.341	
		No	31.05 (23.84–38.26)	1		
**D15S117**	Responder	Yes	n.r.	1.37 (0.40–4.75)	0.615	0.955
		No	n.r.	1		
	Non-responder	Yes	21.90 (8.70–35.11)	1.32 (0.75–2.34)	0.341	
		No	33.80 (5.95–61.65)	1		

OS: Overall survival; CI: confidence interval; n.r.: not reached; * Univariable Cox regression analysis; ** Interaction between response and AI by Cox regression analysis.

**Table 5:** Multivariable Cox regression model.

HR		95% CI	*p*-Value *
**yUICC stage**			0.014
0, 1, 2	1	-	
3, 4	3.00	1.53–5.88	
**Response**			0.553
Yes	1		
No	1.31	0.54–3.19	
**R-category**			0.020
R0	1		
R1	2.16	1.13–4.15	
**Sex**			0.015
Male	1		
Female	0.38	0.18–0.83	
**AI D6S265**			0.091
No	1		
Yes	1.74	0.91–3.30	
**Interaction (AI D6S265 and** **Response** **)**	0.14	0.03–0.63	0.010

HR: Hazard ration; CI: Confidence interval; * *p*: Multivariable Cox regression model with the included variables: UICC stage 7th edition, R-category, sex, response, AI at D6S265 and the interaction between AI at D6S265 with the response.

## Data Availability

The data presented in this study are available in this article or Supplementary Materials.

## Supplementary Materials

The following supporting information can be downloaded at: https://www.mdpi.com/article/10.3390/cancers15030771/s1, Supplementary Methods. Table S1: Chemotherapy regimens; Table S2: Primer Sequences; Table S3: Composition of the multiplex PCRs and upper and lower threshold values for the definition of AI; Table S4: AI and clinicopathological characteristics; Table S5: Survival data and AI of all patients (*n* = 158); Table S6: Multivariable Cox regression model; Figure S1: Overview of the study’s enrollment.
