# Supplementary material for: Significant Tumor Regression after Neoadjuvant Chemotherapy in Gastric Cancer, but Poor Survival of the Patient? Role of MHC Class I Alterations
Source: Cancers (Basel). 2023 Jan 26;15(3):771. doi: 10.3390/cancers15030771 (PMC9913563; doi:10.3390/cancers15030771)
Supplement: Supplementary file 1 [file cancers-15-00771-s001.zip › cancers-2082217-supplementary-final version.pdf]

cancers

MDPI

Supplementary Materials: Significant Tumor Regression after Neoadjuvant Chemotherapy in Gastric Cancer, but Poor Survival of the Patient? Role of MHC Class I Alterations

Theresa Hiltner, Noémi Szörenyi, Meike Kohlruss, Alexander Hapfelmeier, Anna-Lina Herz, Julia Slotta-Huspenina, Moritz Jesinghaus, Alexander Novotny, Sebastian Lange, Katja Ott, Wilko Weichert and Gisela Keller

1. Supplementary Methods

1.1. Design and cycling conditions of the multiplex PCRs

Multiplex Manager 1.2 was used to virtually design multiplex PCRs [36]. Amplification was performed using a Type-It Microsatellite PCR Kit (Qiagen, Hilden, Germany). DNA (20 ng) was added to each PCR reaction at a final volume of 25 µl. The cycling conditions were as follows: after an initial step of 95°C for 5 min, 32 cycles were performed consisting of denaturation at 95°C for 30 s, annealing at 59°C (markers for chromosome 6p21) or 60°C (markers for chromosome 15q21) for 90 s, extension at 72°C for 30 s, and final extension at 60°C for 30 min.

1.2. Determination of individual cut-off values for the definition of AI

Determination of the individual cutoff values for definition of AI for each microsatellite marker was according to previous publications [21,37]. In brief, DNA from 56 non-tumorous tissues from 10 patients was amplified. The range of variation of the amplification of the alleles of each marker was determined by dividing the allele ratio (peak area of the shorter allele divided by the peak area of the longer allele) of the heterozygous markers for each sample. Individual cutoff values were calculated as described in detail previously using the lower and upper bounds (2.5% and 97.5% quantiles) of the bootstrapped two-sided 95% confidence intervals (CI), respectively [37,22]. The lower and upper threshold values for the definition of AI for the microsatellite markers used in this study are included in Supplementary Table S3. A representative dot plot of the amplification variation is shown in Figure S2.

Only informative tumors, which means only patients with a heterozygous genotype for the respective marker, could be evaluated. AI values were calculated by dividing the allele ratio of the normal DNA by the matched tumor DNA [21,38]. Only tumors without microsatellite instability were included in the AI determination. Examples of electropherogramms of tumors with and without allelic imbalances at markers on chromosome 6p21 and 15q21 are shown in Figure S3.

2. Supplementary Tables

Table S1. Chemotherapy regimens.

Neoadjuvant chemotherapy	*n%*	
Total	158	100
Cis + 5-FU	119	75.3
Ox + 5-FU	36	22.8
Ox + Cap	3	1.9

Cis, cisplatin; Ox, oxaliplatin; 5-FU, 5-fluorouracil; Cap, capecitabine;.

Table S2. Primer Sequences.

Marker	Forward Primer	Reverse Primer	Size (bp)	Ref.

**D6S291**	[FAM]CTCAGAG-GATGCCATGTCTAAAATA	GGGGATGACGAATTATTCAC-TAACT	198-210	[39]
**D6S273**	[HEX] GCAACTTTTCTGTCAATCCA	ACCAAACTTCAAATTTTCGG	130-140	[39]
**D6S265**	[FAM] ACGTTCGTACCCATTAACCT	ATCGAGGTAAACAGCAGAAA	122-138	[40]
**D6S2872**	[HEX] CACAGCAGGAAAGGGTTGAC	CCATGAAAAAGTCTGTCCCG	150	[41]
**D15S508**	[FAM] CTCTGAACTTACCATGCTGG	CAGTGTAAACATTACCCCCA	159	[42]
**D15S1028**	[HEX] TGTCCTGAAATTCCCAAC	GAACTGTGCTCTGTCTC	171-187	[31]
**D15S119**	[FAM]AACAGAAAATCCG-TAACATAACATA	ACTTTTGTGCCATTTAGAGATT	185-197	[40]
**D15S982**	[FAM] ATGTTTAAATTAATAAC-GTGACAGT	GACTTCATCTGGATTCACAA	110-138	[34]
**D15S117**	[HEX] GCACCAACAACTTATCCCAA	CCCTAAGGGGTCTCTGAAGA	132-150	[40]

Table S3. Composition of the multiplex PCRs and upper and lower threshold values for the definition of AI.

**Multiplex PCR**	**Marker**	**Primer [µM]**	**Lower threshold value**	**Upper threshold value**
HLA complex region chromosome 6p21	D6S291	2	< 0.74	> 1.39
	D6S273	2	< 0.65	> 1.24
	D6S265	2	< 0.66	> 1.26
	D6S2872	2	< 0.80	> 1.18
B2M region chromosome 15q21	D15S508	0.25	< 0.80	> 1.28
	D15S1028	2	< 0.84	> 1.19
	D15S119	1	< 0.83	> 1.16
	D15S982	0.5	< 0.81	> 1.20
	D15S117	2	< 0.79	> 1.17

Table S4. AI and clinicopathological characteristics.

	**p-values ***							
**Marker**	**Age / median**	**Sex male / female**	**Laurén histotype intestinal / non-intestinal**	**Localisation proximal / non-proximal**	**ypT 0,1,2 / 3,4**	ypN negative / positive	Metastasis status negative / positive	Resection category R0 / R1
**D6S291**	0.698	0.344	0.694	0.841	0.097	0.045	0.671	1.000
**D6S273**	0.251	1.000	0.706	0.121	0.841	0.845	1.000	1.000
**D6S265**	0.569	0.065	1.000	0.421	0.552	0.564	1.000	0.618
**D6S2872**	0.106	0.801	1.000	0.055	0.286	0.687	1.00	0.799
**D15S508**	1.000	0.767	1.00	0.242	0.587	0.430	0.782	0.019
**D15S1028**	0.846	0.346	0.560	0.008	0.686	0.421	1.000	0.623
**D15S119**	0.316	0.340	0.070	0.023	0.056	0.840	0.830	1.000
**D15S982**	1.000	1.000	1.000	0.169	0.552	0.121	0.834	0.624
**D15S117**	0.112	0.216	0.601	1.000	0.358	0.722	0.324	0.824

* Chi-squared or Fisher exact test.

Table S5. Survival data and AI of all patients (n=158).

Marker	AI status	OS median (months) (95% CI)	HR (95% CI)	p- value*
**D6S291**	yes	37.90 (0.00 – 82.34)	0.88 (0.52-1.51)	0.652
	no	61.20 (26.75-95.65)	1	
**D6S273**	yes	66.00 (20.78-111.22)	1.06 (0.63-1.77)	0.826
	no	57.80 (30.92-84.69)	1	
**D6S265**	yes	38.03 (10.63-65.43)	1.25 (0.74-2.12)	0.397
	no	66.00 (49.14-82.86)	1	
**D6S2872**	yes	31.34 (16.31-46.38)	1.65 (0.93-2.91)	0.087
	no	66.10 (47.86-84.34)	1	
**D15S508**	yes	61.2 (15.28-107.12)	1.00 (0.47-2.11)	1.000
	no	44.62 (2.13-87.16)	1	
**D15S1028**	yes	62.23 (20.16-104.30)	1.09 (0.63-1.88)	0.769
	no	66.10 (38.70-93.50)	1	
**D15S119**	yes	66.00 (36.11-95.90)	0.83 (0.48-1.42)	0.498
	no	31.90 (20.61-43-12)	1	
**D15S982**	yes	48.10 (20.26-75.94)	1.15 (0.68-1.93)	0.599
	no	36.6 (1.48-71.72)	1	
**D15S117**	yes	31.34 (9.86-52.83)	1.63 (0.97-2.72)	0.063
	no	69.93	1	

AI, allelic imbalance; OS, overall survival; HR, hazard ration; CI, confidence interval. * p univariable Cox regression model

3. Supplementary Figures

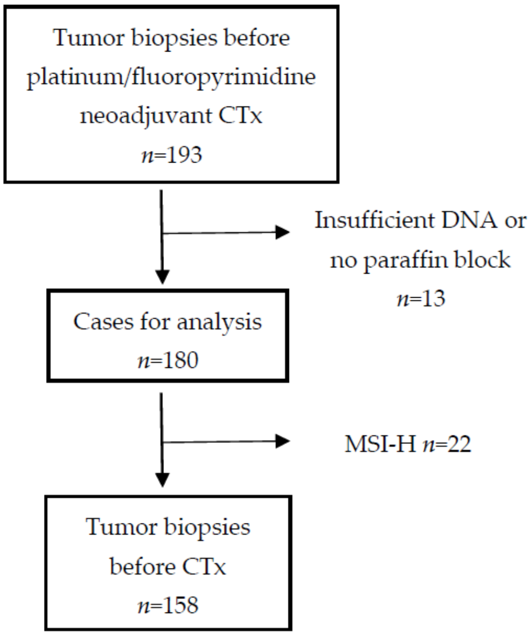

Figure S1. Overview of the study enrolement.

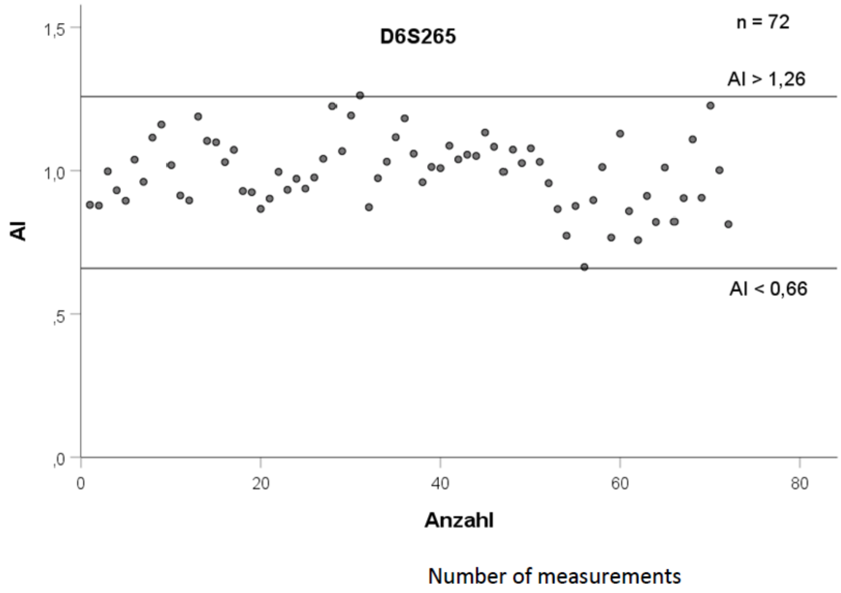

Figure S2. Dot plot of the AI values of marker D6S265 for the cut-off determination. Representative example of a dot plot showing the range of variation of amplification for marker D6S265. Variation was determined by dividing the allele ratio (peak area of the shorter allele divided by the peak area of the longer allele) of the heterozygous markers for each sample. Individual cutoff values were calculated using the lower and upper bounds (2.5% and 97.5% quantiles) of the bootstrapped two-sided 95% confidence intervals (CI), respectively and are indicated by a line.

a

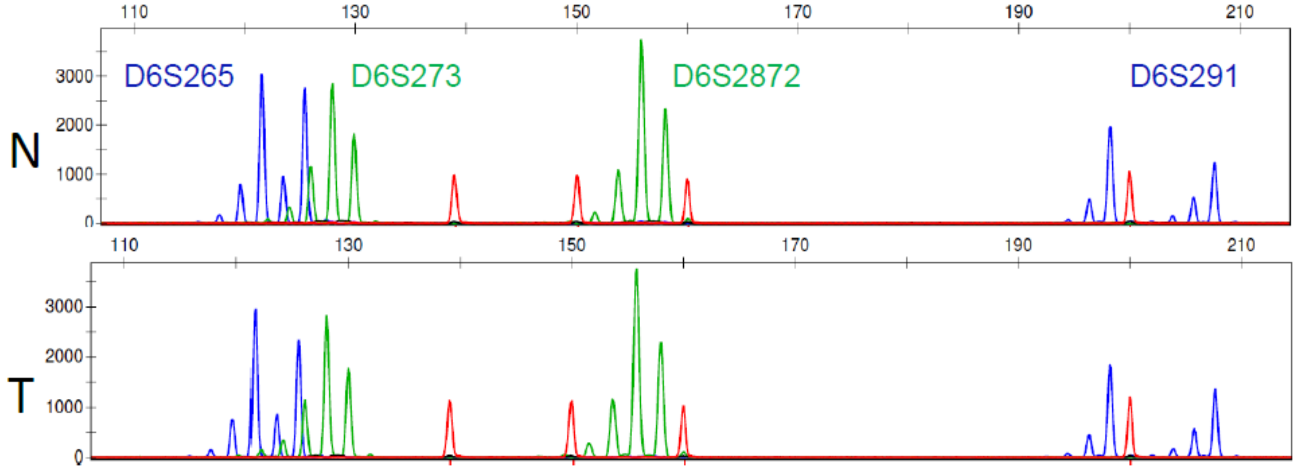

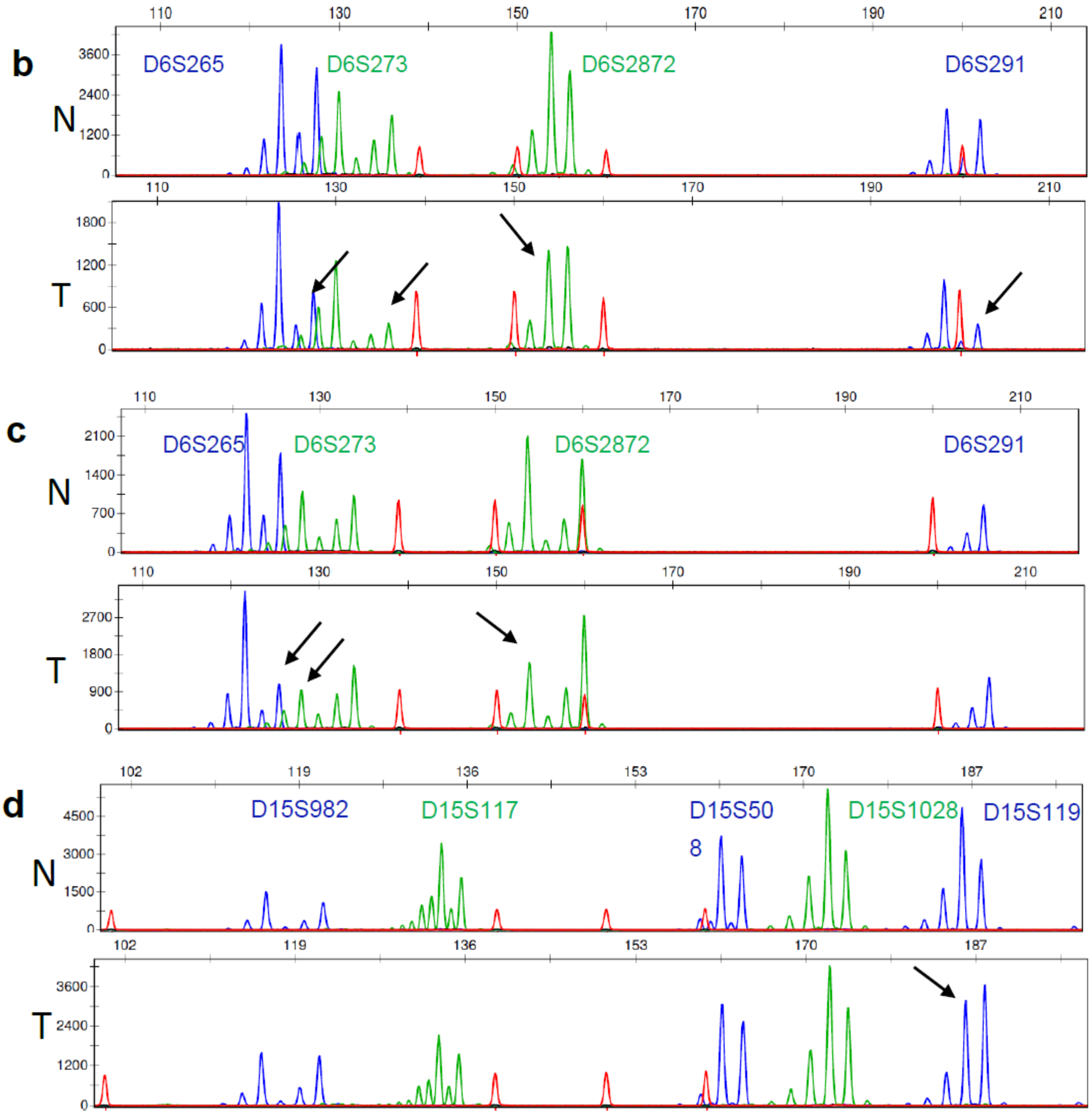

Figure S3. Capillary electrophoresis results of allelic imbalance using microsatellite markers amplified by multiplex PCRs. Electropherogramms of the microsatellite patterns are shown exemplarily for multiplex PCRs of tumor specimen (T) and the corresponding non-tumorous tissue (N) of the four microsatellite markers spanning chromosome 6p21 (D6S291, D6S273, D6S265, D6S2872) (a – c) and of the five microsatellite markers spanning chromosome 15q21 (D15S508, D15S1028, D15S119, D156S982, D15S117). Examples of tumors without allelic imablance (a) and with allelic imbalances (arrows) at chromosome 6p21 (b,c) and at chromosome 15q21 (d) are shown. FAM (blue) and HEX (green) refer to the fluorescence-tagged fragments of the respective microsatellite marker. The red peaks are internal size standards. The x-axis displays the size in bases and the y-axis the respective fluoresence intensity.
